# Immunoglobulin G4-related disease in the stomach presenting as a gastric subepithelial tumor: Case report

**DOI:** 10.1097/MD.0000000000022078

**Published:** 2020-09-04

**Authors:** Min Ji Cho, Hee Seok Moon, Hyeon Seok Lee, Jae Ho Park, Ju Seok Kim, Sun Hyung Kang, Eaum Seok Lee, Seok Hyun Kim, Jae Kyu Sung, Byung Seok Lee, Hyun Yong Jeong

**Affiliations:** Division of Gastroenterology, Departmentof Internal Medicine, Chungnam National University Hospital, Chungnam National University School of Medicine, Daejeon , Republic of Korea.

**Keywords:** asymptomatic subepithelial tumor, gastrointestinal tract, immunoglobulin G4-related disease

## Abstract

**Introduction:**

Immunoglobulin G4-related disease (IgG4-RD) is an immune-mediated fibroinflammatory disorder characterized by specific pathologic findings and often, but not in all cases, elevated serum IgG4 concentration. Although it can virtually involve every organ system, cases involving the gastrointestinal tract and especially gastric mass lesions have rarely been reported.

**Patient concerns:**

A 45-year-old man, who was incidentally discovered asymptomatic subepithelial tumor (SET), by endoscopy, on the greater curvature of the upper gastric body, was referred to our hospital for further evaluation.

**Diagnosis:**

The patient was postoperatively diagnosed with IgG4-RD by histopathologic results.

**Interventions:**

The patient underwent laparoscopic wedge resection.

**Outcomes:**

The patient is presently followed up annually in our clinic and had no problems and showed no signs of recurrence in examination.

**Conclusion:**

We reported a rare case of IgG4-RD presenting as a gastric SET. The first line treatment of IgG4-RD is glucocorticoid administration. However, because pathologic examination is challenging owing to the lesion location, preoperative diagnosis is difficult and may lead to unnecessary gastric resection. Thus, using alternative preoperative diagnostic methods such as endoscopic ultrasound-guided fine-needle biopsy or the biopsy unroofing technique could spare the patient from unnecessary surgical treatment.

## 1 Introduction

Immunoglobulin G4-related disease (IgG4-RD) is an immune-mediated fibroinflammatory disorder characterized by specific pathologic findings such as dense lymphoplasmacytic infiltration of IgG4-positive plasma cells, storiform fibrosis, obstructive phlebitis, and often, but not in all cases, elevated serum IgG4 concentrations.^[1]^ IgG4-RD was not recognized as a systemic disorder until 2003. In 2001, Hamano et al^[2]^ first reported autoimmune pancreatitis exhibiting high serum IgG4 concentration termed “sclerosing pancreatitis,” and in 2003, Kamisawa et al^[3]^ proposed a novel clinicopathological entity using the term IgG4-RD.

Although the pathogenesis of the disease remains unproven, type 2 T-helper cell immune response and regulatory T-cell activation have been suggested to be associated with IgG4-RD.^[1]^ Diagnosis of IgG4-RD can be confirmed by serological, imaging, and histopathological findings.^[4]^ It can virtually involve every organ system: the pancreas, biliary tree, kidneys, retroperitoneum, prostate, aorta, breast, lymph nodes, thyroid, salivary glands, lungs, pericardium, and skin.^[1,5–12]^ The pancreas is the most commonly involved organ; however, cases involving the gastrointestinal tract in particular, cases of gastric subepithelial tumor (SET) caused by IgG4-RD have been rarely reported.

Here, we present a rare case of IgG4-RD in the stomach that was diagnosed as SET by endoscopy. Written informed consent was obtained from the patient for the publication of this manuscript and accompanying images.

## 2 Case presentation

A 45-year-old man, who was diagnosed with gastric SET detected on endoscopy during a routine medical examination, was referred to our hospital in August 2016 for further evaluation. He was symptom-free and presented no specific abdominal symptoms such as pain, vomiting, heartburn, dysphagia, or change in bowel habits. He had no history of medication or family history of malignant disease or autoimmune disorder.

In the physical examination, his blood pressure was 110/70 mmHg and heart rate was 80 bpm. The abdomen was soft and flat, and no palpable mass was observed. His laboratory results were normal for the following parameters: leucocyte count, 8900/mm^3^; hemoglobin level, 14.3 g/dL; and platelet count, 226,000/mm^3^. His liver function tests were normal. Serum IgG4 or other immunoglobulin levels were not measured because there was no reason to suspect IgG4-RD at that time.

**Figure 1:**
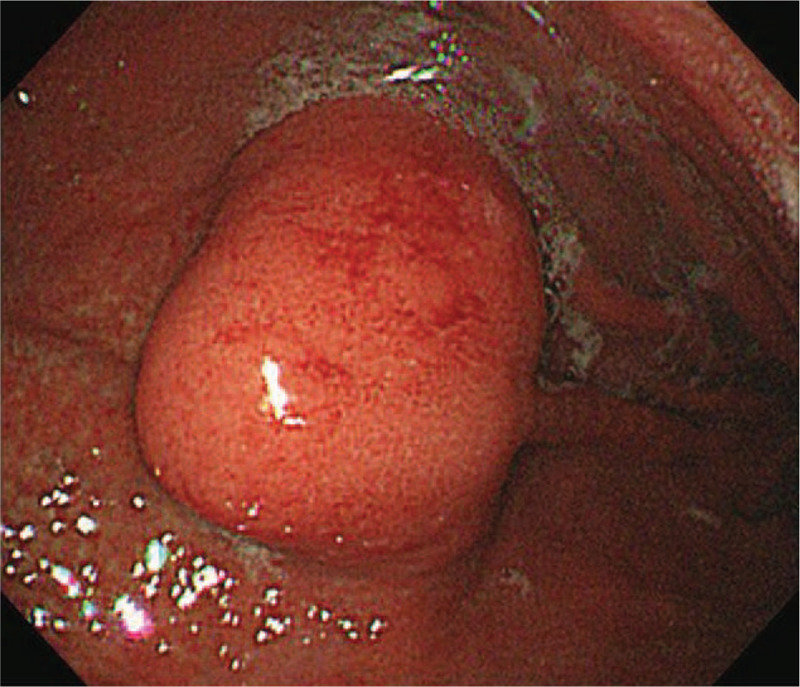
Endoscopy: a 3 × 3 cm-sized fixed, round, and hard submucosal lesion at the greater curvature of the upper gastric body.

**Figure 2:**
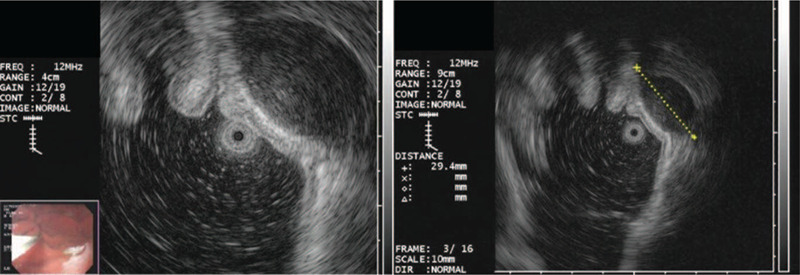
Endoscopic ultrasonography: oval-shaped and hypoechoic intramural lesion developing from the muscularis propria, measuring 29.4 × 16.4 mm at its largest diameter.

**Figure 3:**
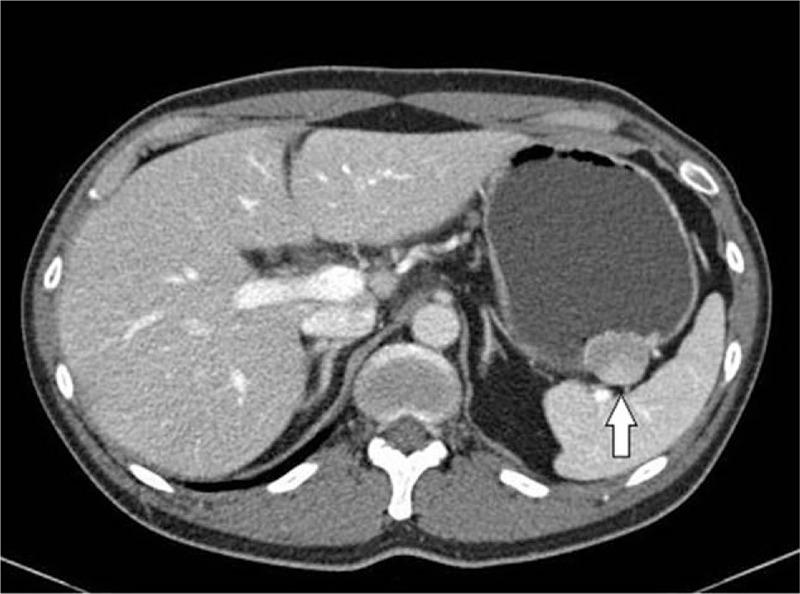
Abdominal computed tomography: a well-defined heterogeneously enhancing wall mass at the upper gastric body.

Endoscopy with endoscopic ultrasonography (EUS) was performed. In endoscopic vision, a 3 × 3 cm fixed, round, hard mucosal lesion was detected on the greater curvature of the upper gastric body (Fig. 1). Endoscopic biopsy showed only mild chronic gastritis. EUS revealed a mainly hypoechoic, oval with sharp margins intramural lesion developing from the muscularis propria and measuring 29.4 × 16.4 mm at its largest diameter (Fig. 2). Abdominal computed tomography (CT) showed a well-defined heterogeneously enhancing wall mass at the greater curvature of the upper gastric body (Fig. 3), most likely to be a malignant gastrointestinal stromal tumor (GIST). No lymphadenopathy was noted.

Laparoscopic wedge resection was performed using Endo GIA^TM^ iDrive purple 60 mm (Covedien, New Haven, CT, USA). The postoperative course was uneventful, and the patient was discharged on the fourth postoperative day.

**Figure 4:**
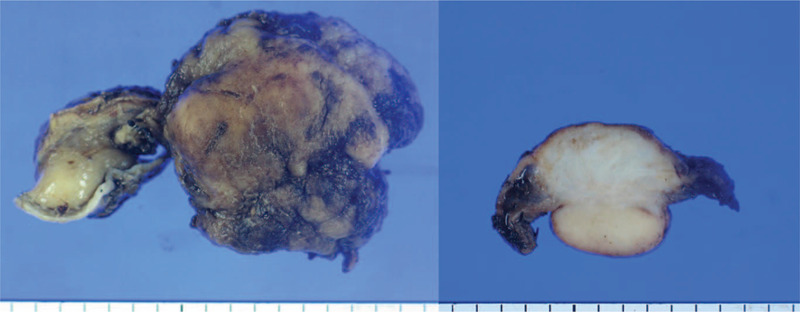
Gross specimen: ill-demarcated tan whitish-colored firm mass and white-gray color round mass in the cut section, which involved the submucosa and subserosa.

**Figure 5:**
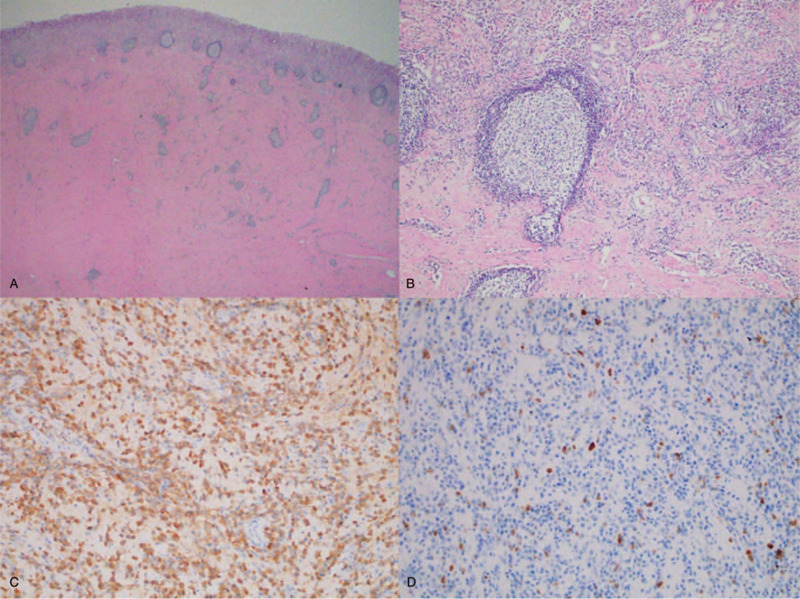
(A) Hematoxylin and eosin (H & E) staining, ×12.5: transmural diffuse fibrosis with dense lymphoplasmacytic infiltration. (B) H & E staining, ×100: storiform fibrosis with numerous plasma cells. (C) Immunohistochemical (IHC) staining, ×200: numerous immunoglobulin-positive inflammatory plasma cells. (D) IHC staining, ×200: numerous immunoglobulin G4-positive plasma cells.

Histopathological examination of the lesion revealed a tan whitish-colored firm mass, measuring 3 × 2.8 cm, protruding from the submucosa and subserosa. On cut section, it appeared white-gray in color (Fig. 4). Microscopically, storiform dense fibrosis and dense lymphoplasmacytic infiltration of plasma cells were identified by hematoxylin and eosin staining (Fig. 5A and B). On immunohistochemical staining, the tumor stained negative for anti c-kit protein and SMA; thus, GIST could be ruled out. Besides, we found IgG and IgG4-positive plasma cells in a ratio of IgG4/total IgG 40%, and a number of IgG4-positive plasma cells at approximately 60/high powered field (HPF), which indicated that the lesion could be an IgG4-related inflammatory mass (Fig. 5C and D).

The patient is presently followed up annually in our clinic. In April 2019, the latest follow-up date, the patient did not have any problems and showed no signs of recurrence on endoscopy and abdominal CT examination.

## 3 Discussion

Here, we reported the case of an asymptomatic man with IgG4-RD presenting as a gastric mass, which was surgically resected but could have been conservatively treated had it been diagnosed preoperatively. IgG4-RD is a multi-organ immune-mediated disease that mimics many malignant, infectious, and inflammatory disorders. This condition links many disorders previously regarded as isolated to single organs such as autoimmune pancreatitis, retroperitoneal fibrosis, Riedel's thyroiditis, and Mikulicz's disease.^[1,13]^ The most common features of this disease include swelling of the involved organ, obliterative phlebitis, elevated serum IgG4 concentration, and IgG4-positive plasma cell infiltration.^[1]^ IgG4 is the least abundant IgG in healthy individuals and typically constitutes under 5% of the total serum IgG. However, high serum IgG4 concentration is not used as a single marker for disease diagnosis as high levels of IgG4 can also be observed in allergic conditions such as allergy, asthma, and eczema.^[14]^

Therefore, IgG4-RD is usually diagnosed based on histopathology. The comprehensive diagnostic criteria for IgG4-RD are divided into three categories: 1) diffuse or localized swelling or masses in single or multiple organs; 2) elevated serum IgG4 concentrations >135 mg/dL, and 3) histopathological findings including marked lymphocyte and plasmacytic infiltration and fibrosis, infiltration of IgG4-positive plasma cells in a ratio of IgG4-positive plasma cells/IgG-positive plasma cell >40%, and >10 IgG4-positive plasma cells/HPF. Patients who fulfil all three criteria have a definite diagnosis of IgG4-RD, those who fulfil the first and third criteria have a probable diagnosis, and those who fulfil the first and second criteria have a possible diagnosis.^[4]^ In the present case, the first and third criteria were used to diagnose the patient with probable IgG4-RD because of the lack of serum IgG4 examination.

Shinji et al first reported IgG4-RD involving the stomach in 2004.^[15]^ Lately, few cases were reported of IgG4-RD in the stomach presenting as a gastric ulcer or mass. Seo et al reviewed six cases of IgG4-RD presenting as a gastric mass, including their own case.^[16]^ In most reported cases, the lesion is surgically resected because of misdiagnosis. In fact, the first line treatment of IgG4-RD is glucocorticoid administration and if needed, administration of glucocorticoid-sparing agents such as azathioprine, mycophenolate mofetil, or methotrexate.^[1,9,13]^ The prognosis of IgG4-RD appears to be good over the short-term with steroid therapy.^[17]^

However, IgG4-RD presenting as gastric SET is difficult to diagnose clinically because of its rarity and difficulty to confirm the pathological results without surgery. The pathogenic tissue is usually located on the submucosal layer or even lower; therefore, diagnosis with endoscopic biopsy forceps is difficult. Furthermore, high tumor mobility makes it hard to approach. For these reasons, if gastric submucosal tumors cannot be distinguished with high confidence, despite the use of EUS, most guidelines strongly recommend resection, especially for tumors larger than 2 cm.^[18–20]^ Although gastric wedge resection is not a radical surgery, it can cause complications such as deformity, which in turn could provoke symptoms such as dyspepsia or gastroesophageal reflux disease. The main limitation of this study is the lack of a serum immunoglobulin test, even when diagnosed postoperatively. Thus, only a probable diagnosis was made in this case.

In conclusion, we reported a rare case of IgG4-RD presenting as a gastric SET. Although the diagnosis of the disease in this case was surgically confirmed, medical treatment such as oral glucocorticoid administration would have been a better option had the disease been diagnosed preoperatively. This case illustrates that an IgG4-RD diagnosis should be considered in the case of gastric masses, and performing EUS-guided fine-needle biopsy or the biopsy unroofing technique could be an option to avoid unnecessary surgical treatment.^[21]^

## Acknowledgments

The authors would like to thank Editage (www.editage.com) for English language editing.

## Author contributions

**Conceptualization:** Hee Seok Moon, Eaum Seok Lee.

**Supervision:** Hee Seok Moon, Jae Ho Park, Ju Seok Kim, Sun Hyung Kang, Eaum Seok Lee, Seok Hyun Kim, Jae Kyu Sung, Byung Seok Lee.

**Validation:** Hyun Yong Jeong.

**Writing – original draft:** Min Ji Cho.

**Writing – review & editing:** Min Ji Cho, Hyeon Seok Lee.
